# Upregulated Collagen COL10A1 Remodels the Extracellular Matrix and Promotes Malignant Progression in Lung Adenocarcinoma

**DOI:** 10.3389/fonc.2020.573534

**Published:** 2020-11-26

**Authors:** Yingkuan Liang, Wenjie Xia, Te Zhang, Bing Chen, Hui Wang, Xuming Song, Zeyu Zhang, Lin Xu, Gaochao Dong, Feng Jiang

**Affiliations:** ^1^ The Affiliated Cancer Hospital of Nanjing Medical University, Nanjing, China; ^2^ Department of Thoracic Surgery, Jiangsu Cancer Hospital, Jiangsu Institute of Cancer Research, Nanjing, China; ^3^ Jiangsu Key Laboratory of Molecular and Translational Cancer Research, Cancer Institute of Jiangsu Province, Nanjing, China; ^4^ Department of Thoracic Surgery, The First Affiliated Hospital of Soochow University, Suzhou, China

**Keywords:** lung adenocarcinoma, COL10A1, DDR2, FAK, metastasis

## Abstract

Collagens are major components of the ECM in various organs, including the lungs. Ectopic expression of collagens can regulate the tumor progression and disease outcome through remodeling of the extracellular matrix (ECM). However, it remains largely unexplored whether collagens are involved in the tumor progression of lung adenocarcinoma (LUAD). Analysis of three LUAD transcriptional expression profiles showed that COL10A1 mRNA expression was up-regulated and associated with poor prognosis. Gain- and loss-of-function studies were performed to observe that up-regulated COL10A1 promotes LUAD cell proliferation and invasion *in vitro* and *in vivo*. In molecular mechanism study, we found that COL10A1 interacts with DDR2 and affects the downstream FAK signaling pathway to regulate LUAD cell progression. The expression of COL10A1 on tissue microarray (TMA) was also measured to explore the association between COL10A1 expression and patient outcome. The results addressed that COL10A1 is up-regulated and positively correlated with lymph node metastasis in lung adenocarcinoma, and the COL10A1 expression is also an independent prognostic factor. In summary, the up-regulated COL10A1 remodels the ECM and the COL10A1/DDR2/FAK axis regulates the proliferation and metastasis of LUAD cells, implying that COL10A1 is a promising therapeutic target and prognostic marker for LUAD patients.

## Introduction

Lung cancer is the most common cause of cancer-related deaths worldwide, with 1.8 million people diagnosed and 1.6 million deaths each year (1). Non-small cell lung cancer (NSCLC) is the major histological subtype of lung cancer and accounts for 85% of patients, and lung adenocarcinoma (LUAD) and lung squamous cell carcinoma (LUSC) are the most common subtypes of NSCLC (2). Approximately 70% of lung cancer patients are diagnosed at an advanced stage when they are no longer eligible for surgery, chemotherapy, and radiotherapy. These patients always have a poor prognosis (3), and the 5-year survival rate of lung adenocarcinoma remains low at around 16%. Therefore, it is essential to understand the molecular mechanism of the underlying pathogenesis in LUAD patients.

In lung adenocarcinoma, there are a large number of essential events in cancer cell progression. Extracellular matrix (ECM) remodeling is the most powerful process. Since the ECM comprises the bulk of the stroma, it is primarily responsible for the increased interstitial tissue pressure and stiff mechanical properties of the stroma. In addition to its mechanical influence, the ECM provides important biochemical and physical cues that promote survival, proliferation, and metastasis (4). The collagen family is one of the most important components of the ECM and the abundant proteins in the body. The ectopic expression of collagen family members can alter the features of epithelial cells and remodel the epithelial cells’ morphology and function. This process enhances cancer cell detachment from the original tumor mass and cell invasiveness, which are necessary for metastasis onset, thus allowing cancer cells to enter the bloodstream or lymphatic flow and colonize distant sites. To clarify the mechanisms of lung adenocarcinoma progression, differentially expressed genes between paired lung adenocarcinoma tissues and adjacent nontumor tissues from three different profiles were identified by comprehensive analyses. We identified two upregulated genes Collagen type X alpha 1 (COL10A1) and Secreted Phosphoprotein 1 (SPP1), and one downregulated genes, Sarcoglycan Gamma (SGCG). COL10A1 was the only gene among the three candidates with a significant prognostic impact. COL10A1 is the alpha chain of type X collagen (5). COL10A1, as a member of the collagen family, was verified to be enrichment in the tumor, and many previous reports have revealed that COL10A1 was upregulated in gastric cancer (6, 7), and breast cancer (8). The expression of COL10A1 is associated with metastasis and poor prognosis. However, COL10A1 was detected highly expressed in the plasma of lung cancer patients (9), the role of COL10A1 in the progression of lung adenocarcinoma is not clear.

In our study, we identified that COL10A1 was ectopically upregulated expressed in LUAD tissues. Patients with higher expression of COL10A1 had a worse outcome due to lymph node metastasis. COL10A1 was been identified promoted the metastasis and proliferation of LUAD cells both *in vitro* and *in vivo*. The mechanistic study demonstrated that COL10A1 could physically interact with DDR2 to maintain the expression level of FAK. Clinically, COL10A1 was demonstrated upregulated in LUAD patients, meanwhile, the expression level of COL10A1 was positive related to lymph node metastasis and poor prognosis. COL10A1 could been recognized as an independent risk factor for the prognosis of LUAD patients. Taken together, the molecular mechanisms underlying COL10A1-enhanced LUAD metastasis were elucidated, providing an understanding of novel diagnostic and therapeutic strategies.

## Materials and Methods

### Patient Samples

Forty tumor tissues and paired peripheral normal lung tissues from LUAD patients who underwent surgery at the Department of Thoracic Surgery, Jiangsu Cancer Hospital (Nanjing, China) were collected and subjected to qRT‐PCR, immunohistochemistry, and immunofluorescence analyses. All tumors and paired normal tissues were confirmed by pathologists. None of the patients received neoadjuvant therapy. The clinical and pathological characteristics of each patient were collected after surgery. This study was approved by the Ethics Committee of Jiangsu Cancer Hospital in accordance with ethical standards. All participants provided written informed consent.

### Cell Culture and Reagents

The human LUAC cell lines A549 and H1299 were purchased from American Type Culture Collection (ATCC) and maintained in RPMI‐1640 (KeyGen, Nanjing, China), except for SPCA‐1, which was maintained in DMEM (KeyGen, Nanjing, China), and both media were supplemented with 10% FBS (Life Technologies) at 37°C in a humidified atmosphere with 5% CO2. The FAK inhibitor PF-562271 (ab141360) was purchased from Abcam (Cambridge, UK). Cells were authenticated by STR analysis at Guangzhou Cellcook Biotech Co., Ltd. (Guangzhou, China) Characterized Cell Line Core Facility within the last three years and routinely tested negative for mycoplasma contamination.

### Western Blotting (WB)

Cells were harvested and treated with lysis buffer (RIPA, KeyGen) on ice, and the protein concentration was determined using a BCA Kit (KeyGen). Comparable amounts of extracts were loaded on SDS–PAGE gels and subjected to electrophoresis. After separation on the gel, proteins were transferred to a PVDF membrane. Membranes were blocked in 2% BSA in TBS-T for 1  h and subsequently incubated overnight at 4°C with antibodies against anti-collagen X (ab58632) and anti-integrin β1 (ab179471) purchased from Abcam (Cambridge, UK). Anti-(FAK) (#13009), anti-P-FAK (#8556S), anti-DDR2 (#12133), anti-E-cadherin (#14472), anti-N-cadherin (#13116), anti-vimentin (#5741), and anti-β-Actin (#3700) were purchased from Cell Signaling Technology (Danvers, MA, USA).

### Quantitative PCR Analysis

Total RNA was isolated using TRIzol reagent (Invitrogen). Quantification of mRNA was carried out using PrimeScript RT Master Mix (cat. #RR036A; Takara). Before calculation using the ΔΔCt method, the levels of GAPDH were used to normalize the relative expression levels of mRNA. The primers are provided in **Table S1**.

### Transwell Migration and Invasion Assay

Cells (4 × 10^4^) suspended in medium without FBS were seeded into Transwell chambers (Costar Corning, Kennebunk, ME, USA) with or without Matrigel (Sigma-Aldrich) coating. The lower chamber contained medium with 10% FBS as a chemokine. Twenty-four hours later, the migratory or invasive cells on the lower surface of the chamber were photographed and counted in 10 random microscopic fields after crystal violet staining.

### Wound-Healing Assay

The transfected cells were cultured in 6-well plates. After the cells reached 90% confluence, a standard 200 μl pipette tip was subsequently utilized to scratch linear wounds. In addition, the cell monolayers were cultivated in FBS-free medium. After scratching, the images of the wound closure were captured at 0 and 24 h.

### Real‐Time Cell Analysis

The CIM-plate16 contains 16 wells, each with a modified Boyden chamber, which can be used independently but simultaneously to measure cell migration in real‐time through 8  μm pores of a polyethylene terephthalate membrane on to gold electrodes on the underside of the membrane using the xCELLigence system (ACEA Biosciences, San Diego, CA, USA). Experiments were set up according to the manufacturer’s instructions with the membrane uncoated (migration) or coated with growth-factor-reduced-Matrigel (invasion) (BD BioSciences, Oxford, UK) (20 μl 1:40 diluted Matrigel per well on the upper surface). The cell index (electrical impedance) was monitored every 30 min for the duration of the experiment. Traces show the average of quadruplicate wells.

### EdU Assay

Cell proliferation was tested by an EdU (5-ethynyl-20-deoxyuridine) assay using the Cell-Light EdU DNA Cell Proliferation Kit (RiboBio, Shanghai, China). H1299 cells (1 × 10^4^) were seeded in each well of 96-well plates for transfection with scramble siRNA (si-scb) or COL10A1 siRNA (si-1). After incubation at 37°C and 5% CO_2_ for 48 h, cells were treated with 50 mM EdU and incubated for another 4 h. Cells were then fixed with 4% paraformaldehyde and stained with Apollo Dye Solution for proliferating cells. Nucleic acids in all cells were stained with Hoechst 33342. The cell proliferation rate was calculated according to the manufacturer’s instructions. Images were taken using a fluorescence microscope (Nikon Instruments Inc., Japan).

### Apoptosis Assay

H1299 and A549 cells were treated with control plasmid or circFndc3b overexpression plasmid for 24 h. Thereafter, cells were subjected to H2O2 insult (100 μm) or hypoxia and serum starvation for 18 h and cells were evaluated for apoptosis by TUNEL staining. Apoptosis was measured with the TMR cell death detection kit (Roche Diagnostics) following the manufacturer’s instructions. Images were taken using a fluorescence microscope (Nikon Instruments Inc., Japan).

### *In Vivo* Animal Model and Growth and Metastasis Assays

For In Vivo metastasis assays, 10 female BALB/c nude mice weighing 18–22  g were randomly assigned to two groups. H1299 cells were prepared as a suspension of 10^6^ cells in 200  μl saline and injected subcutaneously after transfection with sh-scb or shRNA. The tumor size was measured every 2 days with calipers. Four weeks after injection, the mice were sacrificed, and the subcutaneous tumors were isolated and measured.

For *in vivo* metastasis assays, 10 female BALB/c nude mice weighing 18–22  g were randomly assigned to two groups. H1299 cells were prepared as a suspension of 0.4 × 10^6^ cells in 200  μl saline and inoculated into nude mice (five mice per group) through the tail vein after transfection with sh-scb or shRNA. After 4 weeks, the mice were killed, necropsies were carried out, and the lung metastatic nodules were counted. Staining with H&E confirmed that the nodules were metastatic tumors. The protocol used for these studies was approved by the Institutional Animal Care and Use Committee of the Affiliated Cancer Hospital of Nanjing Medical University. The animal study was carried out according to the State Food and Drug Administration of China regulations on animal care. Animals were sorted only by treatment, and no exclusion or inclusion of an animal was predetermined.

### Coimmunoprecipitation (co-IP) Analysis

Co-IP assays used an antibody specific for anti-collagen X (ab58632), and the antibody was purchased from Abcam. Briefly, cells were washed with ice-cold PBS and lysed in 500 μl of co-IP buffer (20 mM Tris-Cl, pH 7.5, 150 mM NaCl, 1 mM ethylenediaminetetraacetic acid, 0.5% NP-40, and 5 μg/ml aprotinin). Equal amounts of protein were incubated with 5 μg of primary antibody and 50 μl of protein A-Sepharose at 4°C for 4 h. The protein bound by protein A-Sepharose beads (Santa Cruz Biotechnology) was released and detected by western blot.

### Fluorescence Immunocytochemical Staining

Cancer cells were grown on coverslips, incubated with 5% milk for 1 h, and treated with antibodies specific for anti-E-cadherin (#14472), anti-N-cadherin (#13116), and anti-vimentin (#5741) at 4°C overnight, and these antibodies were purchased from Cell Signaling Technology (Danvers, MA, USA). Then, coverslips were treated with Cy3-conjugated goat anti-rabbit IgG (1:1,000 dilution) and DAPI (300 nmol/L) staining. The images were photographed under a Nikon A1Si laser scanning confocal microscope (Nikon Instruments Inc., Japan).

### IHC Staining

A tissue microarray was obtained from Outdo Biotech Co., Ltd. (Shanghai, People’s Republic of China). Ninety‐two pairs of LUAC tissues and their paired peripheral normal lung tissues were used to construct the tissue microarray. Tissue sections were deparaffinized and rehydrated with graded alcohol. Endogenous peroxidase activity was blocked by incubation in 3% H2O2. Antigen retrieval was carried out with 0.01  M citrate buffer (pH 6.0) and microwave heat induction. Immunohistochemistry was performed on serial 2.5  μm thick tissue sections from the TMAs or the original blocks. Anti-collagen X (ab58632, Cambridge, UK) was used. Individual specimens were evaluated by two pathologists in a blinded manner, and scores greater than 0.5 were defined as positive expression, and scores less than or equal to 0.5 were defined as negative expressions.

### Statistical Analysis

All statistical analyses were performed with SPSS 25.0 software. Qualitative variables were analyzed by chi-square test or Fisher’s exact test. For continuous variables that obey a normal distribution, Student’s t test is used to compare the differences. Otherwise, variables were compared using a nonparametric test for which there was an abnormal distribution. Differences between groups were compared using analysis of variance (ANOVA) when applicable or a nonparametric test. Correlation analysis was performed using the Pearson correlation coefficient method. ROC curve analysis was performed to estimate the diagnostic sensitivity and specificity. Unless otherwise specified, the results are presented as the means ± standard deviation (SD). All statistical tests were two-sided, and P <0.05 was considered statistically significant.

## Result

### COL10A1 Is Dramatically Elevated in Lung Adenocarcinoma Tissues Compared With Nontumor Tissues and Is Associated With Metastasis and Poor Prognosis

**Figure 1:**
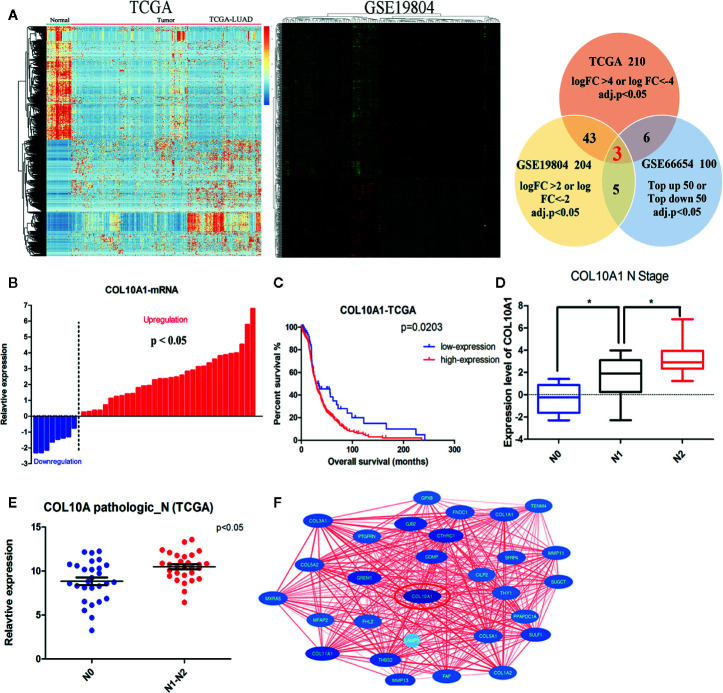
COL10A1 upregulation in lung adenocarcinoma (LUAD) tissues correlates with lymphatic metastasis and poor prognosis in LUAD patients. **(A)** Heatmap of the RNA-seq data of lung adenocarcinoma in the TCGA dataset (left panel) and differential expression in the 60 pairs of nonsmoking women with lung adenocarcinoma based on GSE19804 data (middle panel). Common differentially expressed genes among three datasets are shown in the Venn diagram (right panel) **(B)** The expression of COL10A1 in 40 paired normal and lung adenocarcinoma tissues by qRT-PCR. **(C)** Kaplan–Meier plot analysis of the correlation between the expression of COL10A1 and overall survival in TCGA human lung adenocarcinoma samples. The log-rank test was used. **(D)** Box plots illustrating qRT-PCR analysis of expression fold change for COL10A1 in LUAD tissue with different N stages derived from 40 lung adenocarcinoma tissues and paired nontumor tissues. **(E)** Scatter plots illustrating qRT-PCR analysis of expression fold change of COL10A1 in TCGA LUAD tissues with lymph node metastasis compared with those without metastasis. **(F)** PPI hub networks of COL10A1. Student’s t test and analysis of variance compared the differences in **(D, E**). *P <0.05 vs. N0, N1. The Wilcoxon signed-rank test was used in **(B)**. The log-rank test for survival comparison was used in **(C)**.

To clarify which gene is effective in the malignant progression processes in lung adenocarcinoma (LUAD), we combined and analyzed three transcriptional expression profiles. We performed microarrays to characterize the expression profiles of mRNAs in five paired LUAD tissues and adjacent non-tumor tissues, which has been published before (10), and 100 mRNAs (top-up 50 or top-down 50, P <0.05) were differentially expressed between the lung adenocarcinoma tumor tissues and paired adjacent normal tissues. In addition, we downloaded the data from the Cancer Genome Atlas (TCGA) lung adenocarcinoma database, 210 mRNAs(logFC ≥4 or logFC ≤−4, P <0.05) were identified as differentially expressed (**Figure 1A** left panel and **Supplementary Table S2**). We also selected a Gene Expression Omnibus (GEO) dataset (GSE19804) containing the transcriptional expression profile of 60 pairs of lung adenocarcinoma samples, among which 204 mRNAs (logFC ≥2 or logFC ≤−2, P <0.05) were differentially expressed (**Figure 1A** middle panel and **Supplementary Table S3**). Three differentially expressed genes were common among the three comparisons, they were COL10A1, SPP1, and SGCG (**Figure 1A** right panel). They were all validated in 40 paired LUAD tissues and adjacent normal tissues by qRT-PCR. COL10A1 and SPP1 were upregulated in tumor tissues, and SGCG was downregulated. To explore whether their expressions were associated with prognosis in LUAD patients, we analyzed the expression levels of three candidates in TCGA LUAD samples. Kaplan–Meier curves of overall survival (OS) showed that only the expression of COL10A1 was significantly negative related to poor prognostic (**Figures 1B–C** and **Supplementary Figures S1A–C**), which was selected as the candidate gene. Meanwhile, Interrogation of the TCGA Pan-Cancer dataset revealed that COL10A1 is overexpressed across multiple types of human tumors despite different tissue origins (**Supplementary Figure S1D**). Analyzed the qRT-PCR results of 40 LUAD tissue, we demonstrated that the expression level of COL10A1 was positively correlated with lymphatic metastasis (**Figure 1D**). Simultaneously we detected that LUAD patients with lymphatic metastasis had a higher COL10A1 expression level in the TCGA profile (**Figure 1E**). In addition, the express values of COL10A1 from a GEO dataset (GSE19804) were used to construct the co-expression module *via* the WGCNA package tool (11). The turquoise and blue modules were closely associated with the malignant progression of lung adenocarcinoma. GO analysis was conducted for the turquoise and blue module (**Supplementary Figures S1E–F**). The top 10 significantly enriched biological processes of the turquoise module were related to cell adhesion and immune-related signaling pathways, the blue module was related to cell cycle and chromosome segregation signaling pathways. Therefore, we analyzed the WGCNA module and found that COL10A1 was the hub gene, which implied the potential important function in the progression of LUAD (**Figure 1F**). In general, COL10A1 was verified to be upregulated in lung adenocarcinoma and closely associated with lymphatic metastasis and poor prognosis in LUAD patients. Thus, COL10A1 was selected for further study.

### COL10A1 Promotes Metastasis and Proliferation In Vitro

We examined the expression of COL10A1 in LUAD cell lines by qRT-PCR and western blotting (**Supplementary Figures S1G–H**). The results indicated that COL10A1 was weakly enriched in A549 cells and highly enriched in H1299 cells. Using the COL10A1 vector or small interfering RNAs (siRNAs), we successfully overexpressed or knocked down COL10A1 in A549 cells or H1299 cells, respectively (**Supplementary Figure S1I**).

**Figure 2:**
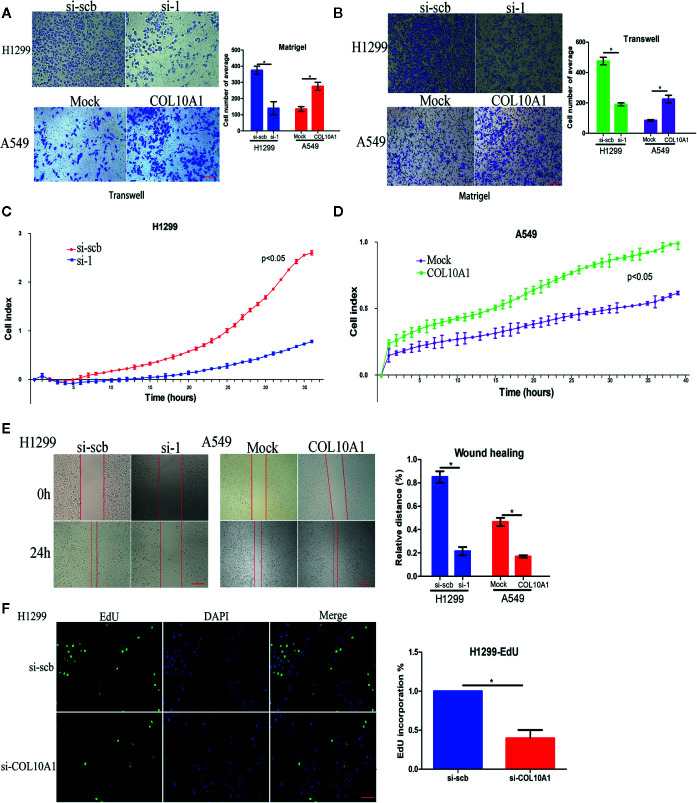
COL10A1 promotes the invasive phenotype *in vitro*. **(A, B)** Representative images (left panel) and quantification (right panel) of Transwell and Matrigel assays showing the invasion of H1299 cells and A549 cells transfected with empty vector (mock), circDCUN1D4, scramble siRNA (si-scb) or COL10A1 siRNA (si-1) (mean ± SD, n = 4). Scale bar: 100 μm. **(C, D)** Cell migration in real-time was analyzed by the xCELLigence RTCA (mean ± SD, n = 4). **(E)** Representative images of the wound healing assay showing the migration of H1299 cells and A549 cells transfected with empty vector (mock), circDCUN1D4, scramble siRNA (si-scb) or COL10A1 siRNA (si-1) (mean ± SD, n = 4). Scale bar: 100 μm. **(F)** An EdU assay was performed to examine the proliferative ability of H1299 cells transfected with scramble siRNA (si-scb) or COL10A1 siRNA (si-1) (mean ± SD, n = 4). Scale bar: 100 μm. Student’s t test and analysis of variance compared the differences in **(A–F)**. ^*^P < 0.05 vs. si-scb and mock.

To further explore the biological function of COL10A1 in LUAD, Transwell chamber migration assays (**Figure 2A**), invasion assays (**Figure 2B**) and real-time cell analysis (RTCA) assays (**Figures 2C, D**) were used to clarify the effect of COL10A1 on metastasis. The migration and invasion abilities were markedly increased upon COL10A1 was overexpressed in A549 cells and inversely reduced when COL10A1 was knocked down in H1299 cells. The same results were observed in wound healing assays (**Figure 2E**). Meanwhile, the EdU assay revealed a significant downregulation in growth ability after transfection with si-COL10A1 compared with the negative control (si-scb) (**Figure 2F** and **Supplementary Figure S2A**). Further, we also observed that knockdown of COL10A1 promoted the apoptosis of H1299 cells in TUNEL assay. In contrast, apoptosis was suppressed in COL10A1-overexpressing A549 cells (**Supplementary Figures S2B–C**). Consequently, it was shown that COL10A1 dramatically increased the malignant cellular-biological behavior of LUAD cells.

### COL10A1 Promotes Lung Adenocarcinoma Metastasis and Growth *In Vivo*

**Figure 3:**
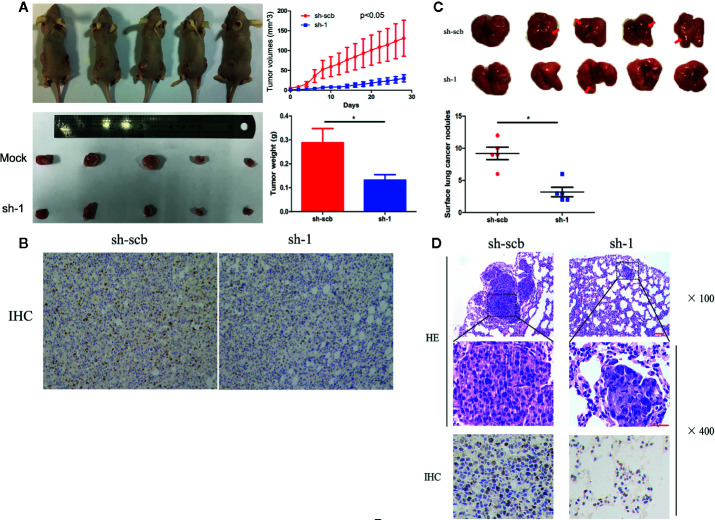
COL10A1 promotes lung adenocarcinoma tumorigenesis *in vivo*. **(A)** Representative images (left panel), *in vivo* growth curve (upper right panel) and weight at the end points of xenograft tumors (lower right panel) formed by subcutaneous injection of H1299 cells transfected with scramble shRNA (sh-scb) or COL10A1 shRNA (sh-1) into the dorsal flanks of nude mice (n = 5 for each group). **(B)** Representative images of immunohistochemical staining revealing the expression of COL10A1 within xenografts formed by subcutaneous injection of H1299 cells transfected with scramble shRNA (sh-scb) or COL10A1 shRNA (sh-1) into the dorsal flanks of nude mice. Scale bar: 100 μm. **(C)** Representative images (upper panel) and surface lung cancer nodules (lower panel) of lung metastatic colonization in nude mice treated with tail vein injection of H1299 cells transfected with scramble shRNA (sh-scb) or COL10A1 shRNA (sh-1) into the dorsal flanks of nude mice (n = 5 for each group). **(D)** Representative images of immunohistochemical staining revealing the expression of HE (upper panel) and COL10A1 (lower panel) within xenografts formed by tail vein injection of H1299 cells transfected with scramble shRNA (sh-scb) or COL10A1 siRNA (sh-1) into the dorsal flanks of nude mice. Scale bar: 100 μm. Student’s t test and analysis of variance compared the differences in **(A, C)**. *P < 0.05 vs. sh-scb.

To explore the effects of COL10A1 *in vivo*, we established a nude mouse subcutaneous xenograft model by implanting H1299 cells with stably transfected with sh-scb and sh-COL10A1. The tumor volumes were monitored for four weeks after H1299 cell injection. The results shown that knockdown of COL10A1 drastically decreased the tumor growth of H1299 cells. The growth rates of the tumors, in terms of volume and weight were decreased upon COL10A1 knockdown (**Figure 3A**). Immunohistochemical staining of subcutaneous xenografts showed the significant decrease of COL10A1-positive cell numbers in COL10A1-knockdown xenografts (**Figure 3B**). In addition, nude mice were treated with tail vein injection of H1299 cells with sh-scb and sh-COL10A1. In the sixth week, mice injected with COL10A1 knockdown H1299 cells showed fewer metastasis nodules than those injected with sh-scb in lung (**Figures 3C, D**). And the fewer COL10A1-positive cells in metastasis nodules of COL10A1-knockdown H1299 cells. These data indicated that COL10A1 exerted an oncogenic role by promoting tumorigenesis and aggressive behaviors.

### COL10A1 Drives Cancer Metastasis *via* Interacting With DDR2 to Facilitate the Phosphorylation of FAK

**Figure 4:**
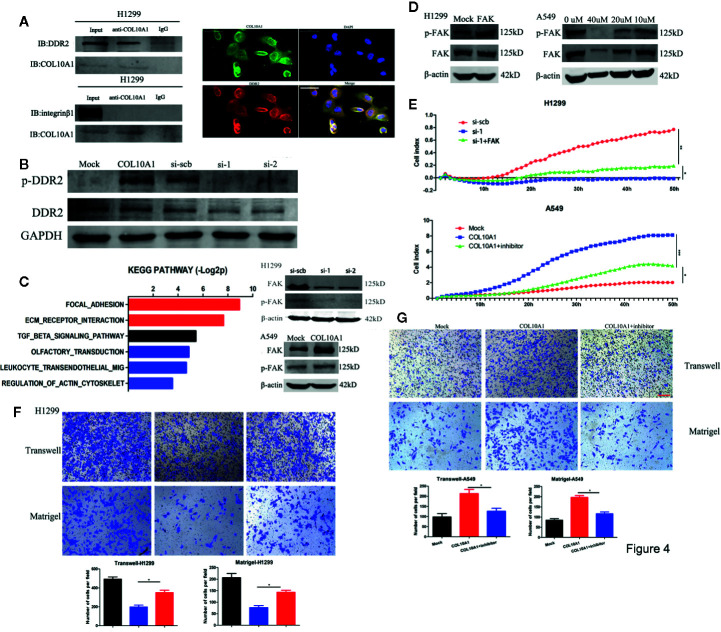
COL10A1 interacts with DDR2 to activate the FAK pathway. **(A)** Co-IP and western blot assays indicating the interaction between COL10A1 and DDR2 but not integrin β1 in H1299 cells (left panel). Immunofluorescence staining assay showing the colocalization of COL10A1 (green) and DDR2 (red) in H1299 cells, with nuclear staining with DAPI (blue) (right panel). Scale bar: 50 μm. **(B)** Western blot assays indicating that COL10A1 promotes the activation of DDR2. **(C)** KEGG pathway analysis of COL10A1 based on the TCGA dataset (left). Representative images of western blots revealing the expression of p-FAK and FAK derived from H1299 cells transfected with scramble siRNA (si-scb) or COL10A1 siRNA (si-1) (upper right panel) and A549 cells transfected with mock and COL10A1 (lower right panel). **(D)** Western blot showing the expression of p-FAK and FAK derived from H1299 cells transfected with mock or FAK (left) and A549 cells transfected with FAK inhibitor at gradient concentrations (right). **(E–G)** Real-time cell analysis (RTCA), Transwell assays, and Matrigel assays showed that knockdown of COL10A1 could suppress the metastasis of H1299 cells and that the suppression could be blocked by overexpressed FAK; in addition, the assays showed that overexpression of COL10A1 could promote the metastasis of A549 cells and that the promotion could be blocked by overexpressed FAK (mean ± SD, n = 4). Scale bar: 100 μm. Student’s t test and analysis of variance compared the differences in **(D, E)**. *P < 0.05, **P <0.001 vs. si-scb, si-1, mock, COL10A1.

The collagen family has been verified could regulate cell adhesion by interacting with various receptors, such as integrin α/β, discoidin domain receptors 1/2 (DDR1/DDR2) (12). Upon reviewed the literatures, we found that DDR2 and integrin β1 were reported could interact with COL10A1 (13, 14). To clarify whether DDR2 and integrin β1 also could interact with COL10A1 in LUAD, we performed co-immunoprecipitation (Co-IP) assays using COL10A1 antibody, DDR2 was confirmed that bingding with endogenous COL10A1 in H1299 cells, but integrin β1 could not be pulled down by COL10A1. In addition, the interaction was also identified by immunocytofluorescent double staining for COL10A1 (green) and DDR2 (red) in H1299 cells (**Figure 4A**). To explore whether COL10A1 could activate DDR2 by physical interaction, the western blot assays were applied to detect the expression level of phosphorylated DDR2 (p-DDR2) upon overexpressed and knocked COL10A1 down in A549 and H1299 cells, respectively. The results demonstrated that COL10A1 could interact with DDR2 to phosphorylate DDR2, which was the activated form of DDR2 (**Figure 4B**). KEGG pathway analysis of COL10A1 revealed that focal adhesion was ranked as the most enriched signaling pathway (**Figure 4C**, left panel). The focal adhesion pathway provides strong adhesion to the matrix (15), meanwhile, the signaling emanating from focal adhesion is driven by the focal adhesion kinase (FAK) (16, 17). Previous reports demonstrated that the activation of DDR2 maintained the expression level of FAK (18). Thus, we explored the effect between COL10A1 and FAK in LUAD. The expression level of FAK was significantly decreased upon knocked COL10A1 down in H1299 cells and was dramatically increased with ectopic expression of COL10A1 in A549 cells. In addition, the phosphorylated FAK (p-FAK) was positive related to the expression levels of COL10A1 and FAK (**Figure 4C**, Right panel). Western blot assays were performed to verify the efficiency of COL10A1 overexpression or silencing by the transfection with FAK vector or FAK inhibitor in H1299 cells or A549 cells (**Figure 4D**). Verified whether the role of COL10A1 in malignant progression of LUAD is dependent on the FAK pathway by functional rescue assays, as expected, COL10A1 promoted the metastasis of lung adenocarcinoma cells, and this promotion was rescued by inhibiting the expression of FAK (**Figures 4E–G**), suggesting that the function of COL10A1 was dependent on the FAK pathway. Taken together, these findings suggest that COL10A1 interacts with DDR2 to maintain the expression of FAK and that the oncogenic function of COL10A1 depends on the FAK pathway.

### The Expression of COL10A1 Induces the Epithelial-To-Mesenchymal Transition(EMT)

**Figure 5:**
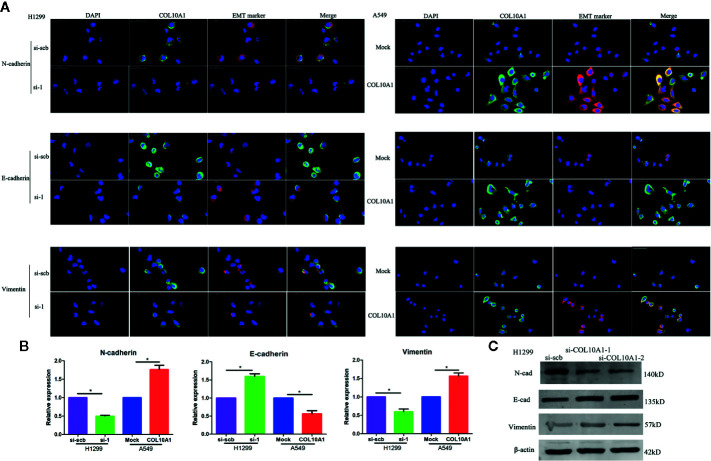
Epithelial–mesenchymal transition (EMT) marker is dramatically affected by COL10A1. **(A)** Immunofluorescence staining assay showing the expression of EMT markers, including N-cad, E-cad, vimentin (red) and COL10A1 (green), in H1299 cells and A549 cells transfected with empty vector (mock), circDCUN1D4, scramble siRNA (si-scb) or COL10A1 siRNA (si-1), with nuclear staining with DAPI (blue) (mean ± SD, n = 4). Scale bar: 50 μm. **(B)** The quantity of N-cadherin, E-cadherin, and vimentin in H1299 and A549 cells, as detected by qRT-PCR. **(C)** The expression of N-cadherin, E-cadherin and vimentin in H1299 cells stably transfected with scramble siRNA (si-scb) or COL10A1 siRNA (si-1) by western blotting. Student’s t test and analysis of variance were used to compare the difference in **(B)**. *P < 0.05 vs. si-scb, mock.

Epithelial-to-mesenchymal transition (EMT) is an important cellular event widely involved in physiological and pathological processes, especially in tumor metastasis (19, 20). The expression of epithelial markers, such as E-cadherin (E-cad), decreases and that of mesenchymal markers, such as N-cadherin (N-cad) and vimentin, increases during EMT progression (21). After transfection of H1299 cells with si-COL10A1 and negative control, immunofluorescence analysis showed that E-cadherin was upregulated, but N-cadherin and vimentin were downregulated (**Figures 5A, B**); the western blot assays revealed the same results (**Figure 5C**). The opposite results were revealed when COL10A1 was ectopically expressed in A549 cells.

We observed that the elevated COL10A1 was significantly induced EMT and promoted the invasive properties and migratory ability of LUAD cells.

### Upregulated COL10A1 Expression Predicts Aggressive Clinicopathological Characteristics and Poor Prognosis in Patients With Lung Carcinoma

**Figure 6:**
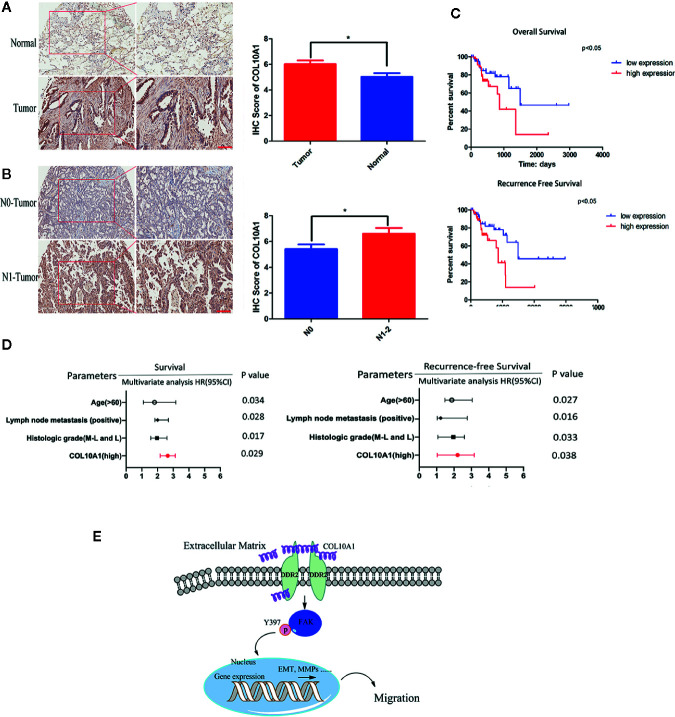
COL10A1 is an independent risk factor for predicting lymphatic metastasis, OS and RFS. **(A)** Representative images of COL10A1 staining in LUAD tissues (left panel) and IHC score of COL10A1 (right panel) in 92 LUAD tissues and their paired normal tissues. Scale bar: 200 μm. **(B)** Representative images of COL10A1 staining in LUAD tissues (left panel) and IHC score of COL10A1 (right panel) in stage N0 and stage N1-2. Scale bar: 200 μm. **(C)** Kaplan–Meier survival curves showed the correlations between COL10A1 expression and OS (upper panel) or RFS (lower panel). **(D)** Multivariate analyses of hazard ratios for OS and RFS showed that COL10A1 is an independent risk factor. **(E)** COL10A1 interacts with DDR2, activates FAK, promotes p-FAK translocation into the nucleus and changes the expression of EMT markers, resulting in the promotion of LUAD metastasis. N1, Metastasis to ipsilateral peribronchial and/or ipsilateral hilar lymph nodes, and intrapulmonary nodes involved by direct extension of the primary tumor; N2, Metastasis to ipsilateral mediastinal and/or subcarinal lymph nodes; OS, overall survival; RFS, recurrence-free survival. Student’s t test and analysis of variance compared the difference in **(A)**. *P < 0.05 vs. tumor, N0. Log-rank test was used for survival comparison in **(C)**.

The expression of COL10A1 was detected by immunohistochemical staining (IHC) in a LUAD tissue microarray (TMA), which contained 92 pairs of LUAD tissues and adjacent normal tissues. Notably, COL10A1 was upregulated in lung adenocarcinoma (**Figure 6A** and **Table 1**). There was a positive correlation between COL10A1 expression and lymph node metastasis (**Figure 6B**). Kaplan–Meier survival curves showed that the LUAD patients with higher COL10A1 expression had poorer overall survival ([OS] p <0.05) and recurrence-free survival ([RFS] p <0.05) (**Figure 6C**). The univariate and multivariate analyses indicated that the COL10A1 expression level was an independent risk factor for OS and RFS, together with age and TNM stage. (**Figure 6D**, **Supplementary Tables S4** and **S5**).

**Table 1:** Correlation between expression of COL10A1 and the clinicopathological features of patients with lung adenocarcinoma.

Variables	Cases(n)(total n = 92)	COL10A1		P value
		Low(n)	High(n)	
Age(yes)				
≤60	43	21	22	0.241
>60	49	18	31	
Gender				
Male	66	30	36	0.343
Female	26	9	17	
Lymph node invasion				
Present	45	6	39	<0.0001*
Absent	47	33	14	
TNM stage				
I	26	21	5	<0.0001*
II-III	66	18	48	
Histologic grade				
High	6	2	4	0.642
Middle-Low	86	37	49	
Smock				
Yes	43	23	20	0.043*
No	49	16	33	

χ2 test was used to test the association between two categorical variables.

*Statistically significant.

Taken together, COL10A1 was upregulated expression in LUAD tissues and related to the lymph node metastasis and the poor prognosis. Meanwhile, COL10A1 was recognized as an independent risk factor for prognosis.

## Discussion

To identify novel tumor biomarkers expressed in lung adenocarcinoma, we assembled microarray datasets from three sources. A bioinformatics approach identified the type X collagen gene (COL10A1) as a gene with elevated expression in lung adenocarcinoma tissues and limited expression in normal tissues. The ectopic expression of COL10A1 is considered to be tightly associated with tumor metastasis (6, 7, 22, 23). In our study, COL10A1 was dramatically upregulated in lung adenocarcinoma tissues compared with adjacent normal tissues, as confirmed by qRT-PCR and IHC. We constructed stable lung adenocarcinoma cell lines with overexpression or knockdown of COL10A1 *via* cell transfection experiments. Through functional experiments *in vivo* and *in vitro*, we found that the invasion and migration abilities of A549 cells overexpressing COL10A1 were significantly upregulated. In contrast, the invasion and migration ability of H1299 cells with COL10A1 knockdown was significantly downregulated. Co-IP and western blotting assays confirmed that COL10A1 could physically interact with the atypical receptor tyrosine kinase DDR2 to elevate the phosphorylation of FAK. The activated FAK signaling pathway plays essential roles in promoting cell migration and invasion (17).

The collagen family is one of the major components of the ECM. The change in collagens can remodel the structure of the ECM, which is a necessary process for tumor metastasis. In high-grade serous ovarian cancer (HGSOC), hypoxic signaling increases the expression of lysyl oxidase (LOX) in mesothelial and ovarian cancer cells to promote collagen crosslinking and tumor cell invasion (24). Metastatic cancers such as bladder, breast, colon, kidney, melanoma, and sarcoma often spread to the lungs, and collagen has been recognized to be involved in this process. During metastasis, cancer cells shape the extracellular matrix (ECM) by hydroxylating collagen. Research indicated that the inhibition of pyruvate metabolism could prevent collagen remodeling in the lung ECM thus reducing metastatic growth in the lung (25).

In lung adenocarcinoma tissues, COL10A1 can originate not only from tumor cells, but also from fibroblasts. Fibroblasts are only modestly enriched in tumors overall, and fibroblasts are responsible for changing the extracellular matrix (ECM) surrounding the tumors. Lung tumors harbor five distinct clusters of fibroblasts, and cluster 1 is the type that is strongly enriched in tumors and exhibits a strong epithelial-mesenchymal transition (EMT) signal. Each fibroblast type expresses a unique repertoire of collagens and other extracellular matrix components, and COL10A1 is the specific high-expressing collagen of cluster 1 in lung carcinoma (26). As members of the collagen family, the origin cells and the special molecular mechanism of COL10A1 upregulation in lung adenocarcinoma are not yet clear, and further studies are required for confirmation.

The collagen family also broadly contributes to cancer development. COL1 and COL5A1 have been reported were ectopically expressed and facilitated the metastasis of LUAD (27, 28). Collagen I, IV and V have been shown to foster adhesion, proliferation, and migration in pancreatic ductal adenocarcinoma (PDAC) (29). Collagen IV-conveyed signals can regulate chemokine production and promote liver metastasis (30). As far as the progression of breast tumors, collagens appear to straighten and align at the tumor-boundary to facilitate the invasion of the tumor into the surrounding stroma (31).The collagen family plays an important role in the invasion and metastasis of tumors

Discoidin domain receptor tyrosine kinase 2 (DDR2) protein is a kind of collagen receptor tyrosine kinase (RTKs) that binds to and is activated by it in the extracellular matrix. Phosphoproteomic research revealed that DDR2 ranked thirteen in RTKs that were highly phosphorylated in a set of 150 NSCLC tumors (32). The DDRs have distinct preferences for certain types of collagens.DDR2 seems to preferentially bind collagen II (33) and collagen X (13).

## Conclusions

In this study, we indicated a critical molecule and biomarker, COL10A1, which was ectopic elevated expression in LUAD. COL10A1 was demonstrated promoted the migration, invasion and proliferation both *in vitro* and *in vivo* of LUAD cells. Mechanistically, COL10A1 in LUAD physically interacts with DDR2 to facilitate the expression level of FAK, aberrant activity of FAK directly promoted the malignant progression of LUAD. Clinically, COL10A1 could as an independent risk factor for OS and RFS. Patients of LUAD with higher COL10A1 expression had a shooter OS and RFS. These findings suggest that COL10A1 acts as a critical prognostic marker for patients with lung adenocarcinoma (**Figure 6E**).

## Data Availability Statement

Publicly available datasets were analyzed in this study. These can be found in The Cancer Genome Atlas (https://portal.gdc.cancer.gov/); the NCBI Gene Expression Omnibus (GSE19804).

## Ethics Statement

This study was approved by the Ethics Committee of Jiangsu Cancer Hospital in accordance with ethical standards. All participants provided written informed consent

## Author Contributions

LX, FJ and GD conceived and designed the experiments. YL and WX designed and carried out most of the experiments, and manuscript writing TZ advised on experimental design and participated in the analysis of data. BC, HW, XS and ZZ helped in cell culture and sample collecting. All authors contributed to the article and approved the submitted version.

## Funding

This study was supported by the grants from the National Natural Science Foundation of China (Grant Nos. 81672294, 81702892), The Project of Invigorating Health Care through Science, Technology Education, Jiangsu Provincial Medical Innovation Team (CXTDA2017002), Funded by Jiangsu Provincial key research development program (BE2017761), The Foundation of Jiangsu Cancer hospital (ZK201601), The young talents program of Jiangsu Cancer Hospital (23), and The Jiangsu Province “Six Talent Peaks Project” (WSN-027).

## Conflict of Interest

The authors declare that the research was conducted in the absence of any commercial or financial relationships that could be construed as a potential conflict of interest.

## Supplementary Material

Supplementary Figure 1
**(A)** The expression of SPP1 in 40 paired normal and lung adenocarcinoma tissues by qRT-PCR(Lift panel), Kaplan-Meier plot analysis of correlation between the expression of SPP1 and overall survival in human lung adenocarcinoma by using TCGA (Right panel). **(B)** The expression of SGCG in 40 paired normal and lung adenocarcinoma tissues by qRT-PCR(Lift panel), Kaplan-Meier plot analysis of correlation between the expression of SGCG and overall survival in human lung adenocarcinoma by using TCGA (Right panel). **(C)** The table showed detail of the three candidate genes. **(D)** High COL10A1 expression is detected across a panel of human tumor types. **(E, F)** Biological process function annotation of turquoise module (Left panel) and blue module (Right panel). **(G)** qRT-PCR showing the expression of COL10A1 cancer cell lines A549, H1975, SPAC-1,H1299 (mean ± SD, n = 4). **(H)** The expression of CLO10A1 in LUAD cell lines by western blot (n = 4 for each group).**(I)** Western blot assay indicating the expression of COL10A1 in H1299 transfected with scramble siRNA(si-scb) or COL10A1 siRNA(si-1,si-2), A549 cells transfected with empty vector (mock), circDCUN1D4, (n = 4 for each group). Student’s t test and analysis of variance compared the difference in I. *P <0.05,**P <0.01 vs. si-scb and mock. Wilcoxon signed-rank test was used in **(A,B)**. Log-rank test for survival comparison in **(A, B)**.

Supplementary Figure 2
**(A)** Edu assay was performed to examine the proliferative ability of COL10A1 in H1299 cells transfected with empty vector (mock) and circDCUN1D4 (mean ± SD, n = 4). **(B, C)** Tunel assay was performed to examine the apoptosis of COL10A1 in H1299 and A549 cells transfected empty vector (mock), circDCUN1D4, scramble siRNA (si-scb) or COL10A1 siRNA (si-1) (mean ± SD, n = 4). Scale bar: 100 μm. Student’s t test and analysis of variance compared the difference in **(A–C)**. *P <0.05 vs. mock and si-scb.

Supplementary Table 2 210 mRNA were identified as differentially expressed genes form the TCGA lung adenocarcinoma database (logFC >4 or logFC <−4, P <0.05).

Supplementary Table 3 204 mRNA were identified as differentially expressed genes form a GEO dataset (GSE19804) (logFC >2 or logFC <−2, P <0.05).

## Abbreviations

NSCLC, Non-small-cell lung cancer; LUAD, Lung adenocarcinoma; LUSC, Lung squamous cell carcinoma; ECM, Extracellular matrix; COL10A1, Collagen type X alpha 1; SPP1, Secreted phosphoprotein 1; SGCG, Sarcoglycan gamma; DDR2, Discoidin domain receptor tyrosine kinase 2; FAK, Focal adhesion kinase; EMT, Epithelial–mesenchymal transition; RTKs, Receptor tyrosine kinases; qRT-PCR, Real-time quantitative polymerase chain reaction; TMA, Tissue microarray.
