# Supplementary material for: Upregulated Collagen COL10A1 Remodels the Extracellular Matrix and Promotes Malignant Progression in Lung Adenocarcinoma
Source: Front Oncol. 2020 Nov 26;10:573534. doi: 10.3389/fonc.2020.573534 (PMC7726267; doi:10.3389/fonc.2020.573534)
Supplement: Supplementary Figure 1 — (A) The expression of SPP1 in 40 paired normal and lung adenocarcinoma tissues by qRT-PCR(Lift panel), Kaplan-Meier plot analysis of correlation between the expression of SPP1 and overall survival in human lung adenocarcinoma by using TCGA (Right panel). (B) The expression of SGCG in 40 paired normal and lung adenocarcinoma tissues by qRT-PCR(Lift panel), Kaplan-Meier plot analysis of correlation between the expression of SGCG and overall survival in human lung adenocarcinoma by using TCGA (Right panel). (C) The table showed detail of the three candidate genes. (D) High COL10A1 expression is detected across a panel of human tumor types. (E, F) Biological process function annotation of turquoise module (Left panel) and blue module (Right panel). (G) qRT-PCR showing the expression of COL10A1 cancer cell lines A549, H1975, SPAC-1,H1299 (mean ± SD, n = 4). (H) The expression of CLO10A1 in LUAD cell lines by western blot (n = 4 for each group).(I) Western blot assay indicating the expression of COL10A1 in H1299 transfected with scramble siRNA(si-scb) or COL10A1 siRNA(si-1,si-2), A549 cells transfected with empty vector (mock), circDCUN1D4, (n = 4 for each group). Student’s t test and analysis of variance compared the difference in I. *P <0.05,**P <0.01 vs. si-scb and mock. Wilcoxon signed-rank test was used in (A,B). Log-rank test for survival comparison in (A, B). [file DataSheet_1.docx]

**Supplementary**

**
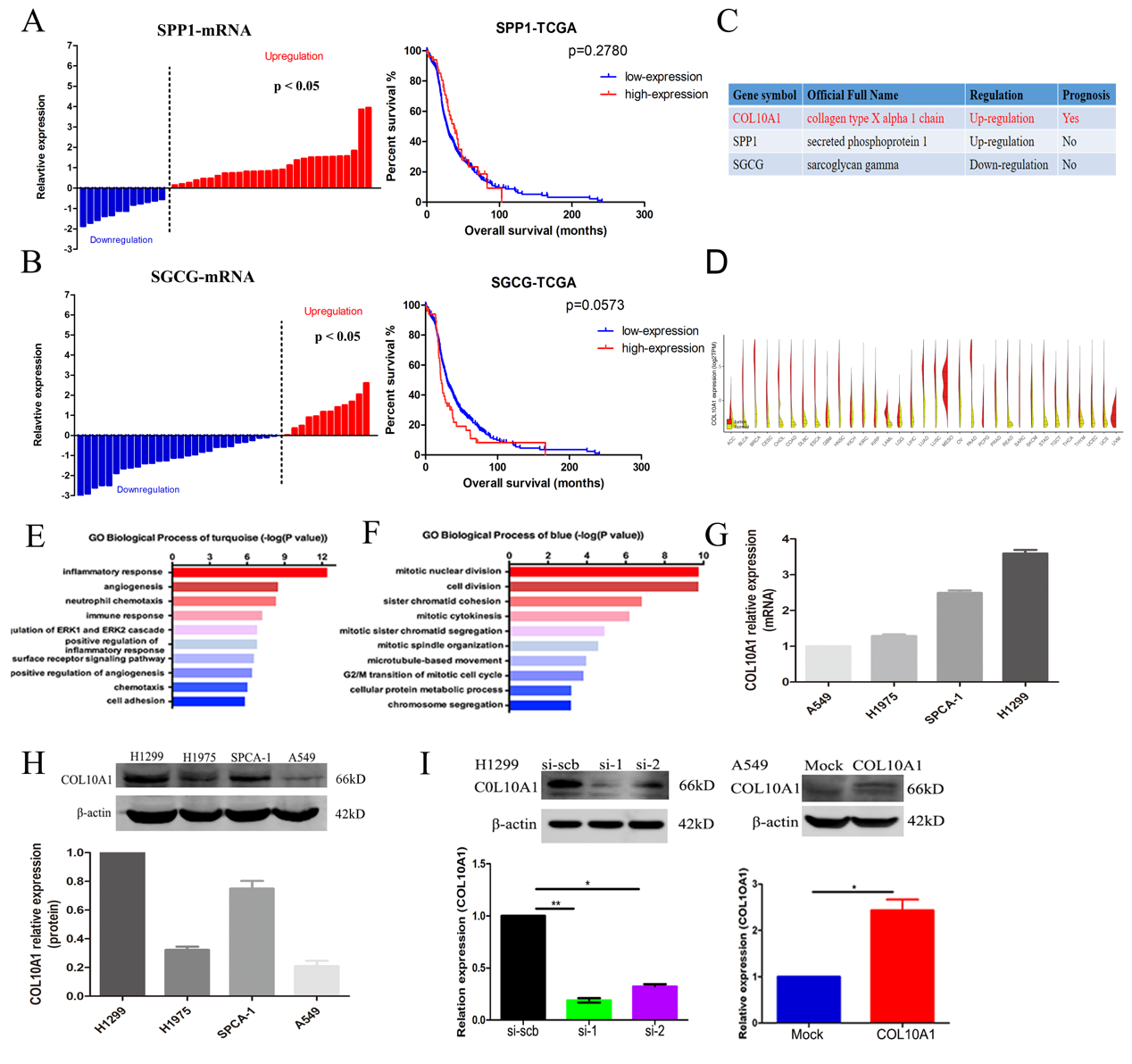
**

**Supplementary Figure 1**

**Fig S1** (A) The expression of SPP1 in 40 paired normal and lung adenocarcinoma tissues by qRT-PCR(Lift panel) , Kaplan-Meier plot analysis of correlation between the expression of SPP1 and overall survival in human lung adenocarcinoma by using TCGA (Right panel).(B) The expression of SGCG in 40 paired normal and lung adenocarcinoma tissues by qRT-PCR(Lift panel) , Kaplan-Meier plot analysis of correlation between the expression of SGCG and overall survival in human lung adenocarcinoma by using TCGA (Right panel). (C)The table showed detail of the three candidate genes. (D) High COL10A1 expression is detected across a panel of human tumor types. (E,F) Biological process function annotation of turquoise module(Left panel) and blue module(Right panel). (G) qRT-PCR showing the expression of COL10A1 cancer cell lines A549, H1975, SPAC-1,H1299 (mean ± SD, n = 4). (H) The expression of CLO10A1 in LUAD cell lines by western blot (n=4 for each group).(I) Western blot assay indicating the expression of COL10A1 in H1299 transfected with scramble siRNA(si-scb) or COL10A1 siRNA(si-1,si-2) , A549 cells transfected with empty vector (mock), circDCUN1D4, (n=4 for each group). Student’s t test and analysis of variance compared the difference in I. *P<0.05,**P<0.01 vs. si-scb and mock. Wilcoxon signed-rank test was used in A,B. Log-rank test for survival comparison in A,B.

**
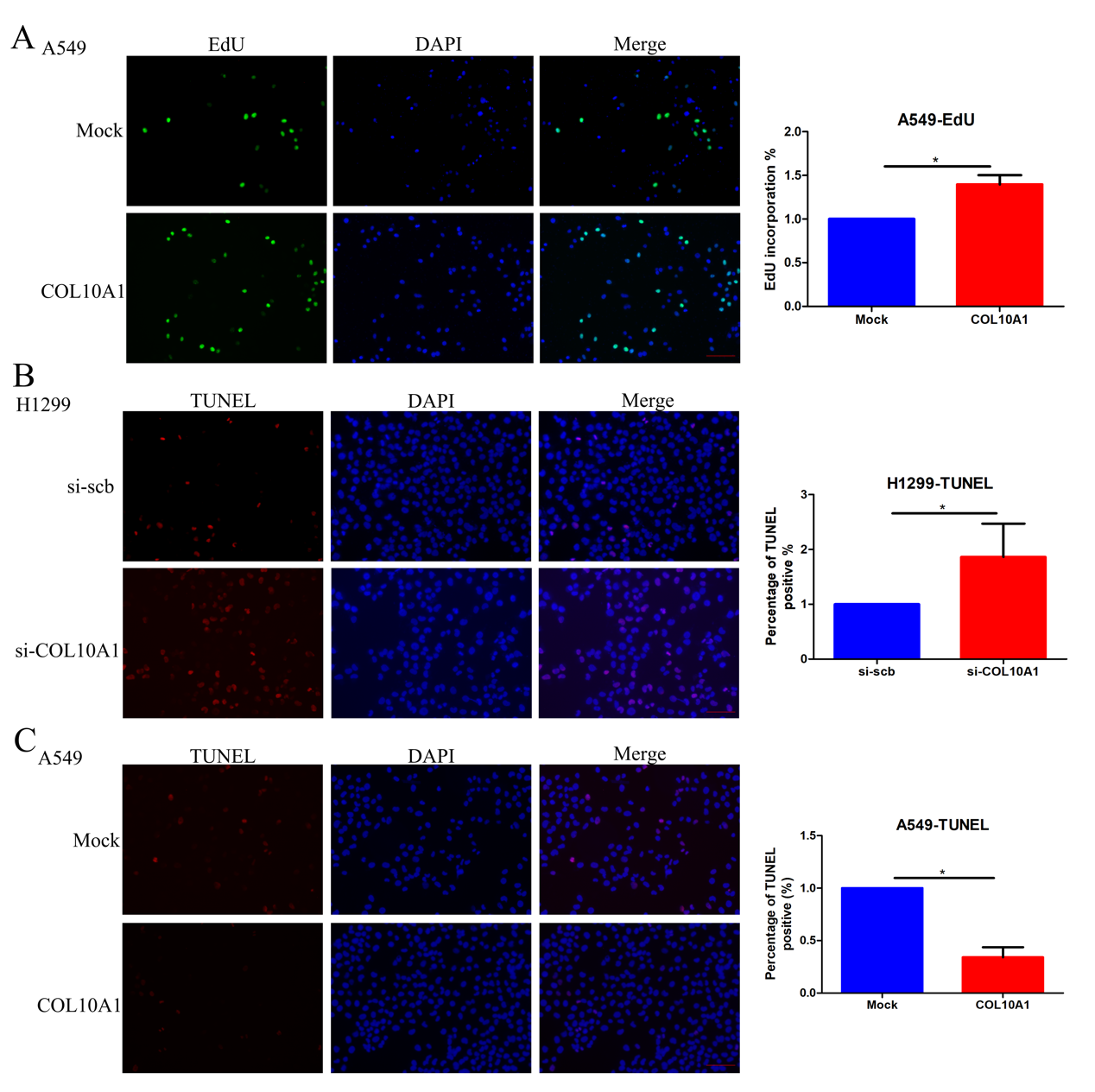
**

**Supplementary Figure 2**

**Fig S2**(A)Edu assay was performed to examine the proliferative ability of COL10A1 in H1299 cells transfected with empty vector (mock) and circDCUN1D4 (mean ± SD, n=4). (B,C)Tunel assay was performed to examine the apoptosis of COL10A1 in H1299 and A549 cells transfected empty vector (mock), circDCUN1D4, scramble siRNA(si-scb) or COL10A1 siRNA(si-1) (mean ± SD, n=4). Scale bar: 100 μm . Student’s t test and analysis of variance compared the difference in A-C. *P<0.05 vs. mock and si-scb.
