# Supplementary material for: Upregulated Collagen COL10A1 Remodels the Extracellular Matrix and Promotes Malignant Progression in Lung Adenocarcinoma
Source: Front Oncol. 2020 Nov 26;10:573534. doi: 10.3389/fonc.2020.573534 (PMC7726267; doi:10.3389/fonc.2020.573534)
Supplement: Supplementary file 2 [file Table_1.doc]

**Supplementary Table S1 Primer sets used forqRT-PCR**

| **Primer set** | **Primers** | **Sequence** | **Application** |
| --- | --- | --- | --- |
| COL10A1 | Forward | 5’-AAGAATGGCACCCCTGTAATGT-3’ | qRT-PCR |
|  | Reverse | 5’-ACTCCCTGAAGCCTGATCCA-3’ |  |
| SPP1 | Forward | 5’-GATGGCCGAGGTGATAGTGT-3’ | qRT-PCR |
|  | Reverse | 5’-GCTTTCCATGTGTGAGGTGA-3’ |  |
| SGCG | Forward | 5’-AACTCGTGAGAGCCCTTTCT-3’ | qRT-PCR |
|  | Reverse | 5’-TACTGCTCACGCACCATCTT-3’ |  |
| GAPDH | Forward | 5’-AGAAGGCTGGGGCTCATTTG-3’ | qRT-PCR |
|  | Reverse | 5’-AGGGGCCATCCACAGTCTTC-3‘ |  |

COL10A1, Collagen Type X Alpha 1 Chain; SPP1, Secreted Phosphoprotein 1; SGCG, Sarcoglycan Gamma;

GAPDH, glyceraldehyde 3-phosphate dehydrogenase.
