# Supplementary material for: Upregulated Collagen COL10A1 Remodels the Extracellular Matrix and Promotes Malignant Progression in Lung Adenocarcinoma
Source: Front Oncol. 2020 Nov 26;10:573534. doi: 10.3389/fonc.2020.573534 (PMC7726267; doi:10.3389/fonc.2020.573534)
Supplement: Supplementary file 5 [file Table_4.docx]

**Supplementary Table4 :Univariate and multivariate Cox regression analyses of overall survival in 92 NSCLC patients**

| Parameters | Univariate analysis | | | Multivariable analysis | | |
| --- | --- | --- | --- | --- | --- | --- |
|  | HR | 95%CI | P value | HR | 955CI | P value |
| Age(>60) | 2.18 | 1.12-3.70 | 0.021 | 1.81 | 1.10-3.16 | 0.034 |
| Gender(male) | 1.56 | 0.60-2.365 | 0.790 | － | － | － |
| Tumor diameter(>3cm) | 2.70 | 1.55-4.96 | 0.670 | － | － | － |
| Pleural invasion(positive) | 1.80 | 1.05-3.90 | 0.083 | － | － | － |
| Lymph node metastasis(positive) | 3.45 | 1.30-4.32 | 0.005 | 2.01 | 1.83-2.69 | 0.028 |
| Histologic grade(M-L and L) | 2.66 | 1.09-3.43 | 0.043 | 1.97 | 1.57-2.60 | 0.017 |
| COL10A1(high) | 3.23 | 1.25-4.43 | 0.016 | 2.65 | 2.17-3.13 | 0.029 |
