# Supplementary material for: Upregulated Collagen COL10A1 Remodels the Extracellular Matrix and Promotes Malignant Progression in Lung Adenocarcinoma
Source: Front Oncol. 2020 Nov 26;10:573534. doi: 10.3389/fonc.2020.573534 (PMC7726267; doi:10.3389/fonc.2020.573534)
Supplement: Supplementary file 6 [file Table_5.docx]

**Supplementary Table5 :Univariate and multivariate Cox regression analyses of recurrence-free survival in 92 NSCLC patients**

| Parameters | Univariate analysis | | | Multivariable analysis | | |
| --- | --- | --- | --- | --- | --- | --- |
|  | HR | 95%CI | P value | HR | 955CI | P value |
| Age(>60) | 1.92 | 1.50-3.03 | 0.042 | 1.87 | 1.47-3.05 | 0.027 |
| Gender(male) | 1.06 | 0.56-3.36 | 0.80 | － | － | － |
| Tumor diameter(>3cm) | 1.113 | 0.53-3.96 | 0.32 | － | － | － |
| Pleural invasion(positive) | 1.63 | 0.90-2.89 | 0.16 | － | － | － |
| Lymph node metastasis(positive) | 1.849 | 1.58-3.97 | 0.001 | 1.23 | 1.03-2.76 | 0.016 |
| Histologic grade(M-L and L) | 2.80 | 1.11-3.66 | 0.001 | 1.96 | 1.07-2.60 | 0.033 |
| COL10A1(high) | 2.62 | 1.64-3.65 | 0.003 | 2.20 | 1.03-3.16 | 0.038 |
